# Drug testing and flow cytometry analysis on a large number of uniform sized tumor spheroids using a microfluidic device

**DOI:** 10.1038/srep21061

**Published:** 2016-02-15

**Authors:** Bishnubrata Patra, Chien-Chung Peng, Wei-Hao Liao, Chau-Hwang Lee, Yi-Chung Tung

**Affiliations:** 1 Academia Sininca, Research Center for Applied Sciences, Taipei, 11529, Taiwan; 2 National Yang-Ming University, Institute of Biophotonics, Taipei, 11221, Taiwan; 3 National Taiwan University, Department of Physics, Taipei, 10617, Taiwan

## Abstract

Three-dimensional (3D) tumor spheroid possesses great potential as an *in vitro* model to improve predictive capacity for pre-clinical drug testing. In this paper, we combine advantages of flow cytometry and microfluidics to perform drug testing and analysis on a large number (5000) of uniform sized tumor spheroids. The spheroids are formed, cultured, and treated with drugs inside a microfluidic device. The spheroids can then be harvested from the device without tedious operation. Due to the ample cell numbers, the spheroids can be dissociated into single cells for flow cytometry analysis. Flow cytometry provides statistical information in single cell resolution that makes it feasible to better investigate drug functions on the cells in more *in vivo*-like 3D formation. In the experiments, human hepatocellular carcinoma cells (HepG2) are exploited to form tumor spheroids within the microfluidic device, and three anti-cancer drugs: Cisplatin, Resveratrol, and Tirapazamine (TPZ), and their combinations are tested on the tumor spheroids with two different sizes. The experimental results suggest the cell culture format (2D monolayer vs. 3D spheroid) and spheroid size play critical roles in drug responses, and also demonstrate the advantages of bridging the two techniques in pharmaceutical drug screening applications.

According to the World Cancer Report 2014, cancers have been identified as the leading causes of morbidity and mortality worldwide, with approximately 14 million new cases and 8.2 million cancer related deaths in 2012^1^. In order to fight the cancer crusade, researchers have been developing various *in vitro* models to better understand fundamental mechanisms of malignant tumor development as well as to test the newly developed therapies^2^,^3^,^4^. In conventional dish-based two-dimensional (2D) monolayer cell culture, cellular activities are often altered and lost their typical *in vivo* functions. As a result, the conventional cell culture provides limited predictive capacity for drug testing^5^. In order to better mimic physiological tissues and further improve the predictive capacity, three-dimensional (3D) cell culture methods have obtained increasing attentions to construct *in vitro* models^3^,^4^. Among 3D cell culture methods, cell spheroids, culture of cell aggregates without any scaffold or physical support, is one of the well-characterized approaches of 3D cell culture models for drug testing^6^. A multicellular spheroid is self-assembled clusters of cell colonies with gradients in nutrients, metabolites, catabolites, and oxygen along the radius, naturally mimicking an avascular solid tumor^7^. Consequently, cell spheroids are concentric arrangement of heterogeneous cell population with different cellular activities, which can reconstitute physiological tumor microenvironments *in vitro* to construct drug testing models with greater predictive capacity^8^.

A number of methods have been developed for cell spheroid experiments^9^. Among them, microfluidics provides a promising technique for spheroid formation and culture platforms due to its desired properties, including: automation, small sample volume and cost effective fabrication. In addition, microfluidics is capable of better controlling flows in spatial and temporal domains, which allows precise and more *in vivo*-like microenvironments to study cell behaviors. Using microfluidic devices, it has been demonstrated that cell spheroids can be cultured in a conventional incubator and used for studying the efficiency of a particular drug for certain periods of time. In recent years, various microfluidic devices have been exploited to test anti-cancer drugs on tumor spheroids *in vitro*^10^,^11^,^12^. For instance, Yu *et al.* developed a droplet-based microfluidic system for multicellular tumor spheroid formation and anti-cancer drug testing^11^. In another device, Ziolkowska *et al.* formed and cultured 3D tumor spheroids for 25 days and studied the effect of anti-cancer drug, 5-Fluorouracil (5-Fu). The device was designed with microwell arrays for spheroid formation. Spheroids of HT-29 human carcinoma cells were cultured for 4 weeks, and the response of spheroids to different concentrations of 5-Fu was observed by measuring variation of the spheroid diameters^12^. Also, Das *et al.* studied the effect of anti-cancer drugs, carboplatin and paclitaxel, on epithelial ovarian cancer spheroids^13^. In order to characterize the chemotherapy response, they analyzed the mortality fraction with vital dyes and confocal microscopy. Kwapiszewska *et al.* developed a microfluidic device with hemispherical microwells for spheroid formation, culture, and drug testing^14^. The cell viability after the drug treatments was characterized by estimating cellular reducing power using a fluorescence dye, alamarBlue, with a microplate reader. Recently, Chen *et al.* used a non-adherent polymer fabrication process to construct a microfluidic spheroid formation platform to characterize the efficacy of photo dynamic therapy (PDT) on 3D cell cultures^15^. Using the platform, the spheroids can be retrieved by peeling off the top layer, which may lead to additional physical damages on the cells, possible contamination, and low harvest efficiency. In the study, the cell viability was estimated by counting tens of fluorescence stained spheroids within the device.

Although the existing techniques are capable of performing tumor spheroid formation, culture, and drug testing, the drug efficiency analysis methods are limited and often require additional processing and instrumentation. Currently, the cell viability analysis of the drug treated spheroids in the microfluidic device mainly relies on imaging analysis of spheroid diameters or fluorescence stained 3D cell spheroids using cytotoxicity assays. However, the diameter measurement is often unreliable due to the possible cell morphological change within the spheroids after drug treatments. Furthermore, the analysis of 3D fluorescence stained spheroid requires sophisticated microscopy to image through the relatively large spheroids, and the imaging process is usually time consuming, which makes the high throughput screening infeasible. The fluorescence dyes also often suffer the difficulty to uniformly diffuse into the center of solid tumor spheroids. Therefore, it is difficult to analyze behaviors of the cells in the center of tumor spheroids, which play critical roles in tumor development and progression. Another broadly used method to analyze cell viability is to characterize cellular reducing power using absorbance or fluorescence dyes. The methods also suffer the diffusion problems that may not be able to provide accurate results, and cannot provide statistical information from individual cells.

In order to overcome the challenges, an integrated approach of improving the analysis of pharmaceutical drug testing on 3D tumor spheroids with well-controlled sizes is highly desired. Among various cell analysis techniques, flow cytometry has exceptional high-speed analytical and physicochemical characterization capabilities. Therefore, flow cytometry has gained widespread use in a variety of medical, scientific, and engineering fields^16^,^17^. Flow cytometry is a technique capable of measuring characteristics of single cells as they flow in a fluid stream through a beam of light. The measured properties include a cell’s relative size, granularity or internal complexity, and fluorescence intensity. As a result, flow cytometry is a useful technique to analyze the cells from the anti-cancer drug treated spheroids. However, a large number of cells (in the order of 1 × 10^6^) are usually required for flow cytometry due to its high throughput screening capability, which is in contradiction to the small volume nature of microfluidics. Consequently, a new type of microfluidic device capable of formation, culture, and cultivation of a large number of tumor spheroids needs to be developed to conduct anti-cancer drug testing and analysis using flow cytometry for practical pharmaceutical applications.

This paper reports an integrated approach combining advantages from microfluidics and flow cytometry to perform drug testing and analysis on a large number of uniform sized tumor spheroids. A two-layered microfluidic device is exploited to form 5000 uniform sized tumor spheroids and perform anti-cancer drugs on the 3D cell culture. The bottom layer of the device is equipped with arrays of cubical cavities as cell culture chambers, and the top layer has a meander shape channel covering all the cell culture chambers for medium delivery and exchange. The tumor spheroid size can be well controlled by designing geometries of the device. The drug treated spheroids can then be harvested from the device and dissociated for flow cytometry analysis using conventional protocols. The operation of the device is simply achieved by manual pipetting without professional training and additional instrumentation. The developed microfluidic device is made of an elastomeric material, polydimethylsiloxane (PDMS) that is broadly used to construct various microfluidic devices for cell culture due to its excellent optical transparency, easy manufacturability, and high gas permeability^18^,^19^.

In the experiments, hepatocellular carcinoma spheroids are formed, cultured, and treated with drugs within the device up to 72 hours. Three anti-cancer drugs, including: Cisplatin, Resveratrol, Tirapazamine (TPZ), and their combinations are tested on the formed spheroids for 48 hours. The spheroids are then harvested from the device, and dissociated to single cells for flow cytometry analysis. The experimental results demonstrate that the developed microfluidic device offers a simple yet powerful tool for formation and culture of uniform-sized spheroids with minimal instrumentation and simple setup. Moreover, the integrated approach provides a promising technique to further study cellular behaviors in single cell resolution in 3D spheroids with aids of flow cytometry analysis. The demonstrated combination of the advanced microfluidic device and the conventional analysis method pave a way for future pharmaceutical drug testing with better understanding of the drug mechanisms in 3D cell culture, and provide *in vitro* models with greater predictive capacity for various cancers and diseases.

## Materials and Methods

### Microfluidic device design and fabrication

**Figure 1:**
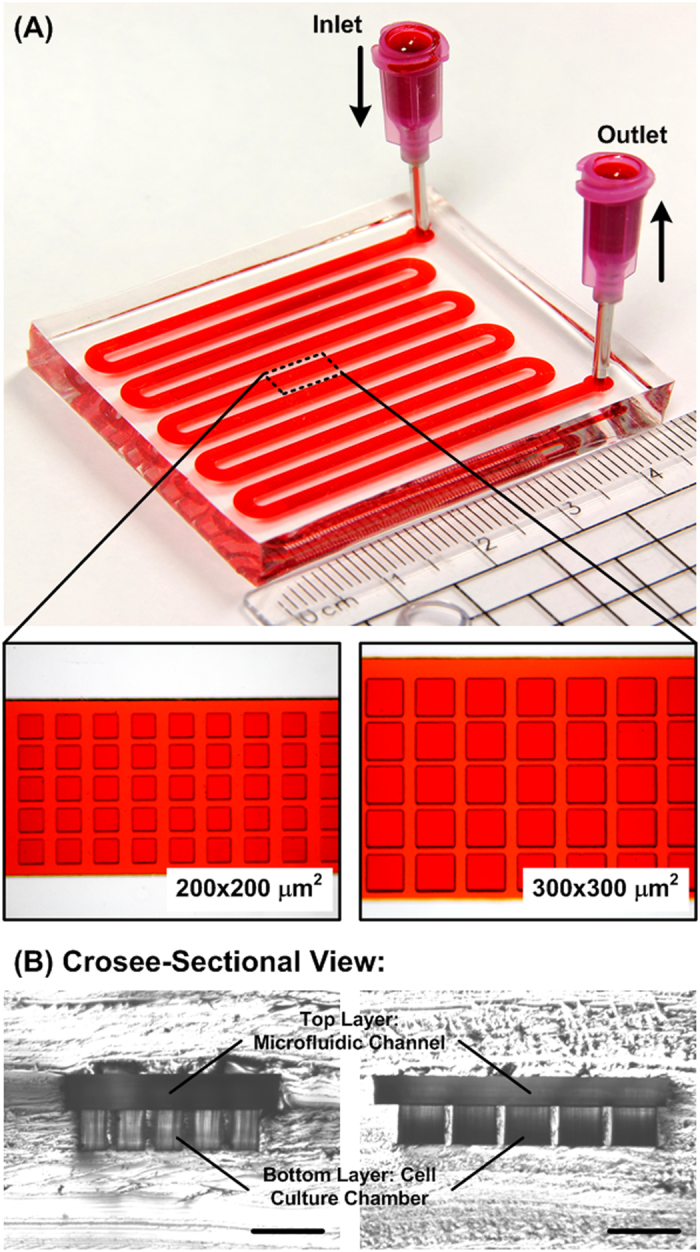
(**A**) Two-layered microfluidic devices for formation, culture, and drug testing of 5000 uniform-sized tumor spheroids with different culture chamber geometries (200 × 200 μm^2^ and 300 × 300 μm^2^) to prepare different sized spheroids. (**B**) Microscopic cross-sectional views of the devices with culture chamber geometries of 200 × 200 μm^2^ and 300 × 300 μm^2^ (width × length), and the height is 250 μm. Scale bar is 500 μm.

The microfluidic device is composed of two PDMS layers: a top layer with a meander shaped microfluidic channel; and a bottom layer with multiple cell culture chambers as shown in Fig. 1. The top layer microfluidic channel covers all the cell culture chambers, and the channel is exploited to deliver cell suspension and exchange medium for spheroid formation, culture, drug testing, and harvesting. The spheroid size can be well controlled by designing the cell culture chamber geometries^18^. The entire device is fabricated using a well-developed soft lithography replica molding process^20^. In brief, silicon wafers with positive relief features are exploited as molds. The molds are constructed by negative tone photoresist (SU-8 2100, MicroChem Co., Newton, MA) patterned by conventional photolithography. The fabricated molds are then silanized with 1H,1H,2H,2H-perfluorooctyltrichlorosilane (78560-45-9, AlfaAesar, Ward Hill, MA) in a desiccator for more than 30 minutes at room temperature to prevent undesired bonding of PDMS to the molds.

PDMS prepolymer (Sylgard 184, Dow Corning Co., Midland, MI) with 1: 10 (v/v) curing agent to base ratio is poured on the molds and cured at 60°C for more than 4 hours. After curing, the interconnection holes are punched using a biopsy punch with a diameter of 1.5 mm at the top layer. The top layer is aligned and irreversibly bonded with the bottom layer using oxygen plasma surface treatment (PX-250, Nordson MARCH Co., Concord, CA) at 90 W for 40 s. The PDMS device is then cured in a 60°C oven for more than 2 hours to promote the bonding and assure full curing of the PDMS to enhance cell compatibility. In the experiments, two sets of devices with different spheroid culture chamber dimensions of 200 × 200 μm^2^ and 300 × 300 μm^2^ (width × length) are designed. The depth of the spheroid culture chambers and top channel height are 250 μm in both sets of the devices. There is a necrotic core when a spheroid is larger than certain size. In order to eliminate the necrotic cell population before the drug testing for better drug response studies, the spheroid culture chamber geometries are designed to form tumor spheroids with sizes that do not show significant necrotic cell population. The footprints of the entire devices are less then 5 × 5 cm^2^ with thickness of 1 cm.

### Cell culture

To demonstrate the device capabilities for tumor spheroid formation, drug testing, and flow cytometry analysis, human hepatocellular carcinoma cells (HepG2, 60025, Bioresource Collection and Research Center, Hsinchu, Taiwan) are utilized for the cell experiments in this study. The stocks of HepG2 cells are cultured in growth medium composed of Minimum Essential Media (MEM) (Gibco 41090-036, Invitrogen Co. Carlsbad, CA) with 10% v/v fetal bovine serum (Gibco 10082, Invitrogen), 1% Antibiotic-Antimycotic (Gibco 15240, Invitrogen), 1% sodium pyruvate (Gibco 11360, Invitrogen) and 1% non-essential amino acids (Gibco 11140, Invitrogen). The cells are maintained under 5% CO_2_ in T25 or T75 cell culture flasks (Nunc 156367, Thermo Scientific Inc., Rochester, NY), and passaged by dissociation with 0.25% trypsin-EDTA (Gibco 25200, Invitrogen) every three days. Cell suspensions for the experiments are made by centrifugation of dissociated cells at 1000 rpm for 5 minutes at room temperature. The culture medium is changed every other day for the cell stocks during the experiments.

### Cell spheroid formation and culture

**Figure 2:**
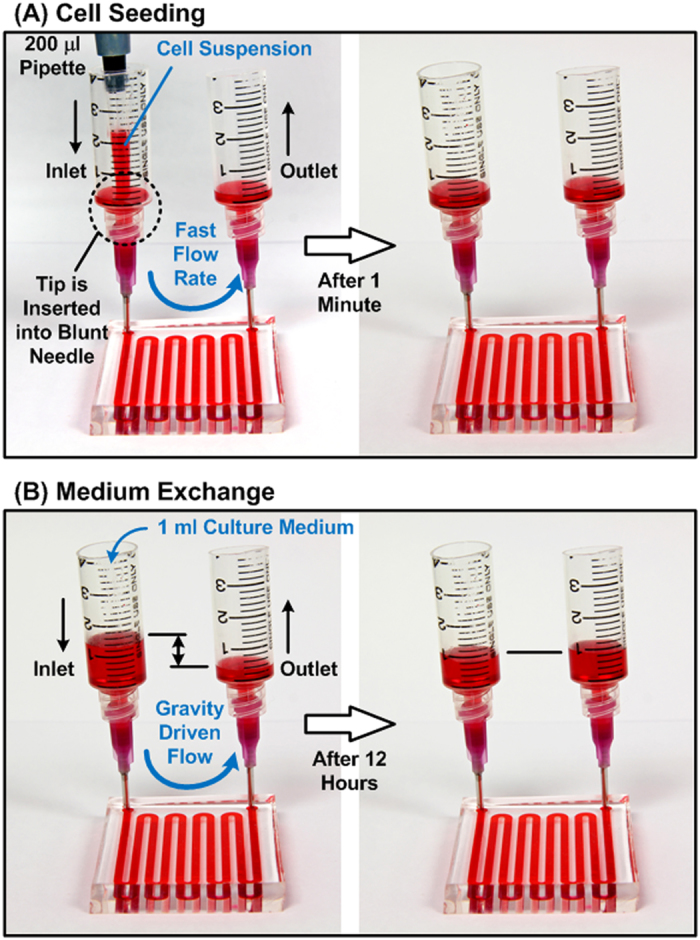
(**A**) Cell seeding procedure: cell suspension is introduced into the device using a 200 μl pipette with a flow rate greater than 100 μl/min to achieve uniform distribution of the cells in the top layer microfluidic channel. The pipette tip is inserted into the blunt needle to assure the fast flow rate inside the device. (**B**) Culture medium exchange procedure: 1 ml of fresh medium (with drug when conducting drug testing) is added from the inlet. The gravity driven flow with a relative flow rate is exploited to replace the aged medium inside the device with minimal disturbance on the spheroids.

Before the cell experiments, the device is oxidized using oxygen plasma (PX-250, Nordson MARCH, Concord, CA) to make the PDMS surface hydrophilic. Afterwards, 1% w/v Synperonic^®^ F-108 (07579, Fluka, SIGMA- ALDRICH, Co., St Louis, MO) is introduced into the channel and incubated overnight in order to make the device surfaces resistant to cell adhesion. The device is sterilized under UV light for 1 hour, and excess Synperonic^®^ F-108 is washed out with the culture medium right before introducing the cell suspension. For spheroid formation, a 170 μl HepG2 cell suspension with density of 2 × 10^7^ cells/ml and a 200 μl cell suspension with density of 6 × 10^7^ cells/ml are introduced into the devices with 200 × 200 μm^2^ and 300 × 300 μm^2^ cell culture chambers, respectively. In order to make the cells distribute uniformly across the entire microfluidic channel in the top layer, the cell suspension is introduced into the device with a flow rate greater than 100 μl/min^17^. After introducing the cells through the microfluidic channel located in the top PDMS layer, the cells docked into the cell culture chambers due to gravity as shown in Fig. 2 (A). After cell seeding, the device is kept in a humidified incubator with 5% CO_2_ at 37 °C. The medium in the device is exchanged every 12 hours by adding 1 ml of fresh culture medium at the inlet and pipetting out the same amount of aged medium from the outlet as shown in Fig. 2(B). In order to characterize the spheroid size distribution within the devices, bright field images of spheroids are captured using an inverted microscope equipped with a CCD camera (ORCA-R2, Hamamatsu Photonics, Shizuoka, Japan) every day.

### Anti-cancer drug testing

In order to demonstrate the device capabilities for drug testing, anti-cancer drugs with different modes of action are tested on the cultured tumor spheroids. In the experiments, three drugs: Cisplatin, Resveratrol, and TPZ are tested on the cells. Cisplatin is a platinum based drug showing clinical activity against wide variety of solid tumors^21^,^22^. Its cytotoxic mode of action is mediated by its interaction with DNA to form DNA adducts, primarily intrastrand crosslink adducts, which activate several signal transduction pathways, and culminate in the activation of apoptosis. The polyphenolic compound Resveratrol is a naturally occurring phytochemical and can be found in many plant species, including grapes, peanuts and various herbs^23^,^24^. Several studies have established that Resveratrol can exert antioxidant and anti-inflammatory activities. It also has activity in the regulation of multiple cellular events associated with carcinogenesis. However, clinical evidence of an effect of Resveratrol on cancers in humans is inconsistent^24^. With a different mode of action, TPZ is an anti-cancer drug activated at low oxygen tensions^25^,^26^,^27^.

In the drug testing experiments, 2D HepG2 cell cultures treated with the three anti-cancer drugs with different concentrations under various oxygen tensions are first performed in well plates. Cell viabilities of the treated cells are estimated by measuring the reducing power of living cells, and the results are shown in Fig. S1 in the supplementary information. For the drug testing on 3D spheroid experiments, the HepG2 cells are introduced into the device to form tumor spheroids for 24 hours before the drug application. From previous studies, the drug testing results suggest that spheroids usually show greater drug resistance^4^,^11^. Therefore, the drug concentrations higher than IC50 on 2D cell cultures are exploited for the spheroid drug testing experiments to demonstrate drug efficiency discrepancy between 2D and 3D cell cultures. Cisplatin (P4394, Sigma-Aldrich), Resveratrol (R5010, Sigma-Aldrich), and TPZ (Toronto Research Chemicals, Inc.) prepared in the HepG2 culture medium with concentrations of 20 μM, 200 μM, and 200 μM are tested on the tumor spheroids for 48 hours, respectively. During the drug testing in the devices, 1 ml of culture medium with the drugs is exchanged using the gravity driven flow every 12 hours. The control experiments are performed with the same protocol without adding drugs. The entire drug testing experiments are also conducted in 2D Petri-dish culture for comparison.

### Flow cytometry analysis

In order to perform the flow cytometry analysis on the drug treated spheroids, the spheroids are harvested out of the devices after the treatments. The spheroids can be flushed out from the spheroid culture chambers by introducing a flow with relatively high flow rate (greater than 1 ml/min) to achieve high harvesting efficiency with great spheroid integrity^18^. The harvested spheroids are then collected from the outlet using manual pipetting, and then dissociated into single cells using trypsin-EDTA 0.25% for 5 minutes with gentle pipetting up and down to minimize aggregated cell population for following analysis (Fig. S2 in the supplementary information). In the experiments, two types of flow cytometry analysis are performed. First, the cell viability assay monitoring cell membrane integrity is exploited to confirm that the spheroids can be formed and well cultured inside the developed microfluidic devices. In the cell viability assay, Calcein AM and 7-Amino-ActinomycinD (7-AAD) dyes are used to stain live and dead cells according to their membrane integrity, respectively. Calcein AM is a cell-permeable dye, and it can be converted to a green fluorescent Calcein from non-fluorescent Calcein AM after acetoxymethyl ester hydrolysis by intracellular esterases within live cells. In contrast, 7-AAD is generally cell-impermeable for live cells, and undergoes a spectral shift upon association with DNA. Therefore, it has been used to label necrotic or late apoptotic/dead cells with damaged cell membranes^28^. The single cell suspension (1.5 × 10^5^ cells/ml) from the dissociated spheroids with volume of 1 ml is incubated with 2 μl Calcein AM (50 μM) (live stain) for 20 minutes at room temperature, protected from light. The suspension is further incubated for 10 minutes with 7-AAD (559925, BD Pharmingen, Becton, Dickinson, and Company, Franklin Lakes, NJ) 5 μl (dead stain) with 1.5 × 10^5^ cells in a 100 μL Dulbecco’s Phosphate-Buffered Saline (DPBS, Gibco 14190, Invitrogen). The stained cells are analyzed by a flow cytometer (BD FACSCalibur) using 488 nm excitation and measuring green fluorescence emission for Calcein AM (530 nm/30 nm band pass) and red fluorescence emission for 7-AAD (650 nm long pass).

Furthermore, apoptosis and necrosis assay based on the expression level of a cellular protein, Annexin V, and cell membrane permeability to 7-AAD is also performed in the experiments to better investigate the anti-cancer drug efficiency and their mechanisms on 3D tumor spheroids. In the analysis, APC-Annexin V (550474, BD Pharmingen) is used to stain the apoptotic cells. Annexin V is a phospholipid-binding protein with a high affinity for phosphatidyl serine (PS). In normal live cells, PS is located on the cytoplasmic surface of the cell membrane. When cells undergo apoptosis, PS is translocated from the inner to the outer leaflet of the plasma membrane, and becomes available for Annexin V binding^29^. As a result, combination of APC-Annxein V and 7-AAD stains can be exploited to distinguish cells undergo necrosis and apoptosis to cell death. After harvesting of the drug treated spheroids, cells are washed twice in cold D-PBS and then re-suspended in 1X Annexin V binding buffer (556454, BD Pharmingen) at a concentration of 1 × 10^6^ cells/ml. A cell suspension of 100 μl is then transferred into 2 ml tube, and a solution of 5 μl of APC Annexin-V and 7-AAD are added to the cell suspension. Cells are incubated for 20 min at room temperature in the dark, and then 400 μl of the binding buffer is added to each tube. All the cell samples are flowed through test tubes with 35 μm nylon mesh cell strainers (Falcon 352235, Corning Inc., Corning, NY) to minimize cell aggregation within the samples for the flow cytometry analysis, and the analysis is performed within an hour. The gating experiments of the flow cytometry analysis are also performed on the same batch of cells without staining and with single stains as shown in Fig. S3 in the supplementary information.

## Results

### Formation and culture of 3D tumor spheroids

**Figure 3:**
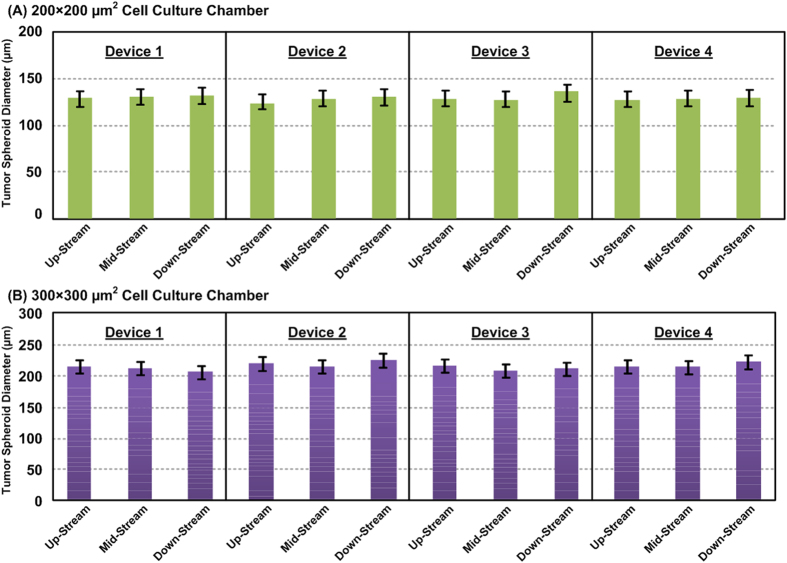
Size distribution analysis of the HepG2 tumor spheroids located in up-, middle- and down-stream of the microfluidic device and different devices with cell culture chamber dimensions (width × length) of (**A**) 200 × 200 μm^2^ and (**B**) 300 × 300 μm^2^, and the height is 250 μm. Data are expressed as the mean ± standard deviation (16 and 9 spheroids are analyzed in each location within a device with 200 × 200 μm^2^ and 300 × 300 μm^2^ cell culture chambers, respectively).

Formation of the tumor spheroids from HepG2 cells occurs spontaneously inside the microfluidic devices as shown in the supplementary information (Fig. S4). The images show that the microscopic images of the cells after seeding into the devices with two different cell culture chamber sizes, and the cells after 1-day static culture. The images suggest that the cells can aggregate and form spheroids after 24 hours due to the strong cell-cell interaction. In order to investigate the spheroid size controllability of the devices, the spheroid size distributions in up-, middle-, and down-stream of devices with different cell culture chamber dimensions are analysed. In addition, the sizes of spheroids formed in four different devices with same geometries are also analysed to estimate variation between the devices. Fig. 3 shows the size distribution of the spheroids in the different regions of the same device and across different devices. The average spheroid diameter formed in 200 × 200 μm^2^ cell culture chambers is approximately 130.5 μm (n = 16) with coefficient of variation (CV, the ratio of the standard deviation to the mean) less than 6%, whereas the average spheroid diameter formed in 300 × 300 μm^2^ chambers is approximately 212.7 μm (n = 9) with CV less than 3%.

### Cell viability assay

**Figure 4:**
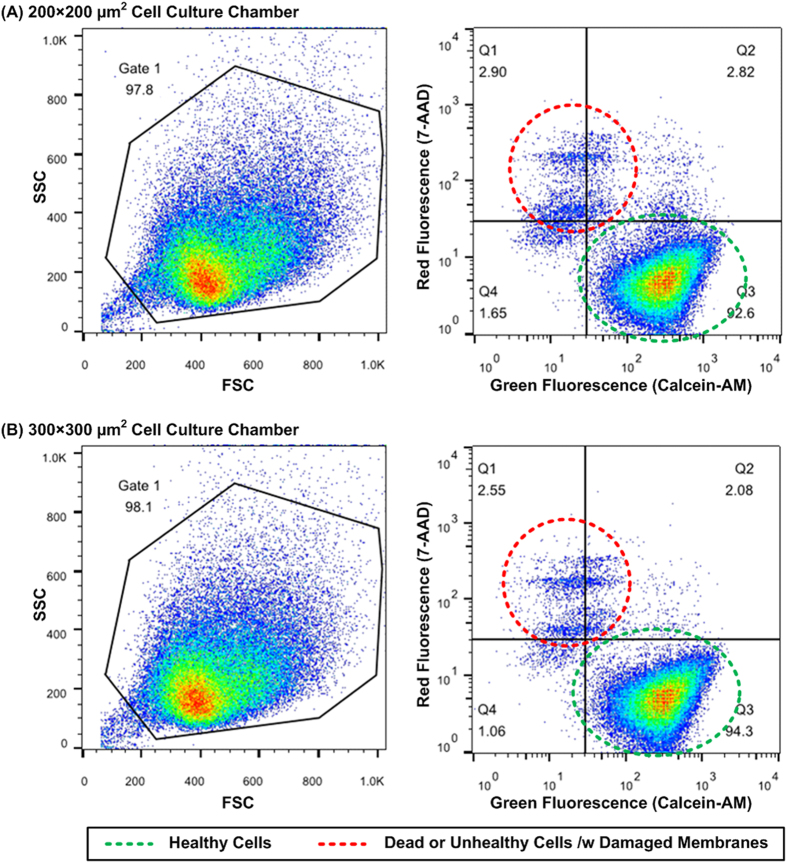
Left: Flow-cytometry analysis of dissociated HepG2 tumor spheroids with forward-scattering light (FSC), side-scattering light (SSC), and gated areas. Right: Viability assay using Calcein-AM and 7-AAD (right panel) on dissociated HepG2 tumor spheroids after 3-day culture in the microfluidic devices with cell culture chamber dimensions (width × length) of (**A**) 200 × 200 μm^2^ and (**B**) 300 × 300 μm^2^, and the height is 250 μm.

Figure 4 shows the flow cytometry viability analysis of HepG2 spheroids cultured for 72 hours in the devices with two different cell culture chamber sizes. The left panel of figure shows the forward-scattered light (FSC) and side-scattered light (SSC) plot with gating to avoid the undesired noises from debris. The right panels show the density plots with green fluorescence intensity (Calcein AM) as x-axis and red fluorescence intensity (7-AAD) as y-axis. The population of live cells labelled by Calcein AM shows at the lower right corner (Q3) in the plot, while the population of dead and unhealthy cells, which lose their membrane integrity, labelled by 7-AAD shows at the upper left part. The density plots obtained from the flow cytometry analysis suggests that 92.6 % and 94.3% of the cells are alive after the 3-day culture inside the microfluidic device.

### Anti-cancer drug testing on 2D and 3D cell cultures

**Figure 5:**
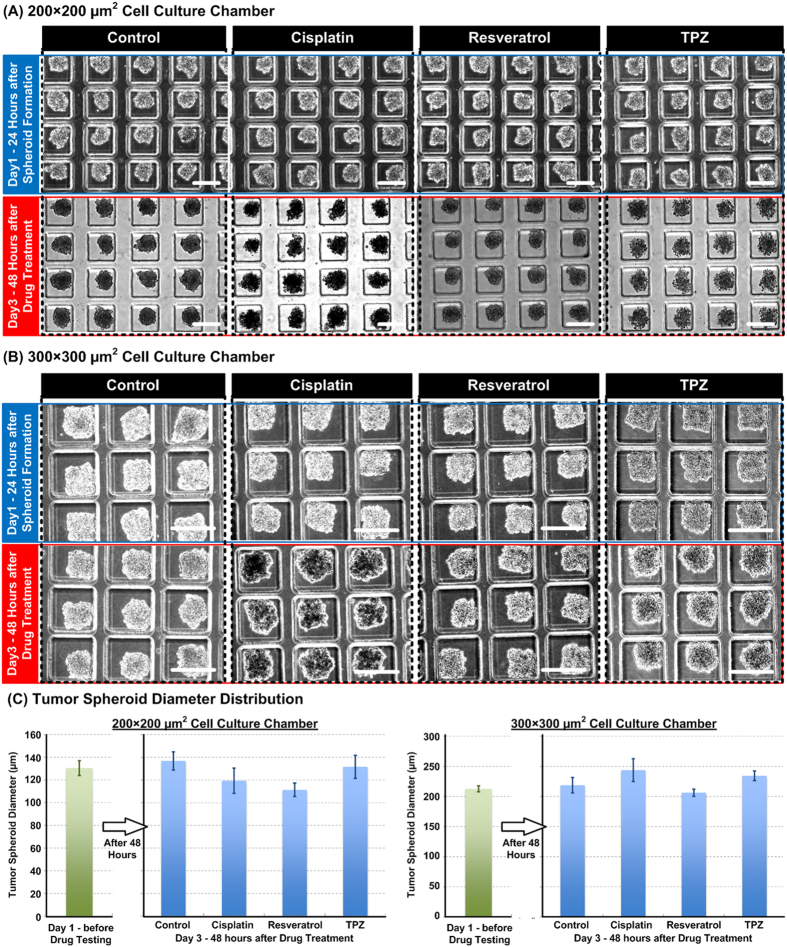
Bright field microscopic images of the HepG2 tumor spheroids cultured in the microfluidic devices with cell culture chamber dimensions (width × length) of (**A**) 200 × 200 μm^2^ and (**B**) 300 × 300 μm^2^, and the height is 250 μm for total 3 days and treated with drugs for 48 hours. (**C**) Quantitative characterization of the diameters of the HepG2 tumor spheroids before and after the drug treatments by imaging analysis on the bright field microscopic images.

Figure 5(A,B) show bright field images of HepG2 spheroids formed in 200 × 200 μm^2^ chambers and 300 × 300 μm^2^ chambers before and after treated with different anti-cancer drugs, respectively. Spheroid diameter has been identified as one of important parameters to characterize drug efficiency when the spheroids undergo drug testing. In order to quantitatively investigate the spheroid size variation under different conditions, we measure the spheroid diameter using the image analysis software, ImageJ (Ver.1.49, National Institutes of Health, Bethesde, MD), on the captured bright field images as shown in Fig. 5(C). The plots show the average spheroid size after 24-hour formation and right before the drug testing, and the average spheroid sizes after 48-hour drug treatments. The results show the average diameters of the spheroids formed in the smaller cell culture chamber increase slightly from average 130.5 μm to 136.8 μm in the control condition without any drug treatment. For the spheroids under drug treatments, their average diameters are all smaller than the control ones after 48 hours. It is noted that the TPZ treated spheroids grow slightly from the average diameter of 130.5 μm to 131.6 μm after the treatment. In contrast, the average diameters of the spheroids formed in the larger cell culture chamber increase slightly from average 212.7 μm to 218.8 μm in the control condition without any drug treatment. After the treatments, both the Cispatin treated spheroids and TPZ treated spheroids have larger average diameters than the control ones, which usually indicates the promotion of cell growth. However, the bright field images as shown in Fig. 5(B) indicate that the Cisplatin treated and TPZ treated spheroids are not as compact as the control ones, suggesting the spheroid integrity may be affected by the drugs and further changes the spheroid sizes. Consequently, the experimental results show that the initial tumor spheroid size is critical for drug responses of the cells, and the spheroid size may not be a reliable parameter to evaluate the drug efficiency.

**Figure 6:**
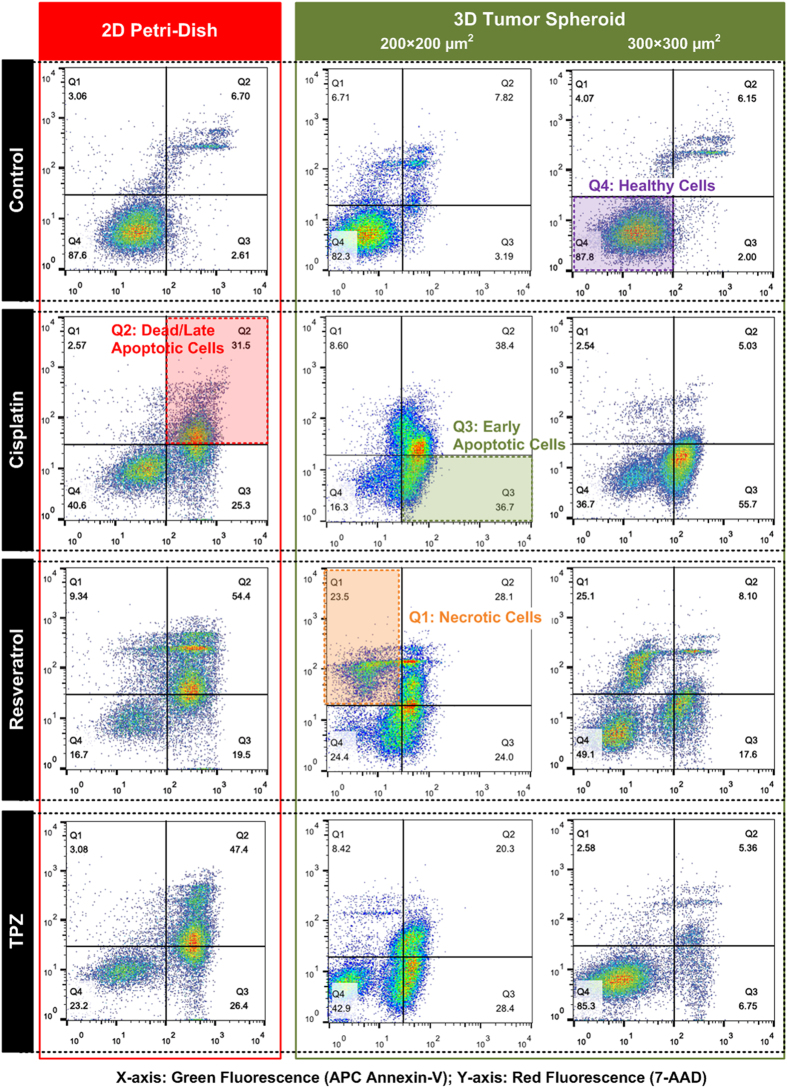
Density plots of flow cytometry analysis on APC-Annexin V and 7-AAD stained HepG2 cells (apoptosis and necrosis assay) cultured in 2D Petri-dish and dissociated from different sized tumor spheroids after 48-hour drug treatments.

In order to quantitatively and precisely analyze the cellular responses after the drug testing, the flow cytometry apoptosis and necrosis assay (Annexin-V and 7-AAD) is performed in the experiments. In the assay, APC Annexin-V stain (green fluorescence) is used to detect the externalization of phosphatidylserine in apoptotic cells, and 7-AAD (red fluorescence) is exploited to stain necrotic and dead cells, which lose their cell membrane integrity. As a result, live cells with intact membranes shown little or no fluorescence; early apoptotic cells show green fluorescence from APC Annexin-V; necrotic cells show red fluorescence from 7-AAD; and dead (late apoptotic) cells show both red and green fluorescence. Figure 6 shows the flow cytometry analysis results on the cells from 2D Petri-dish, and 3D spheroid culture under different anti-tumor drug treatments. The density plots are drawn with green fluorescence intensity (APC Annexin-V) as x-axis and red fluorescence intensity (7-AAD) as y-axis. Therefore, the live cells appear at the lower left corner (Q4) in the plots; the early apoptotic cells appear at the lower right corner (Q3); the necrotic cells appear at the upper left corner (Q1); and dead cells appear at the upper right corner (Q2). In the figure, all the control experiment results show higher than 80% of the cells (87.6 % in Petri-dish, 82.3 % in microfluidic device with 200 × 200 μm^2^ culture chambers, and 87.8% in microfluidic device with 300 × 300 μm^2^ culture chamber) are live with intact cell membranes. In the drug testing on the cells cultured in 2D Petri-dish format, all three drugs show reasonable cytotoxicity and leads more than 30% of cells to late apoptotic stage (32.5% for Cisplatin, 54.4% for Resveratrol, and 47.4% for TPZ).

**Figure 7:**
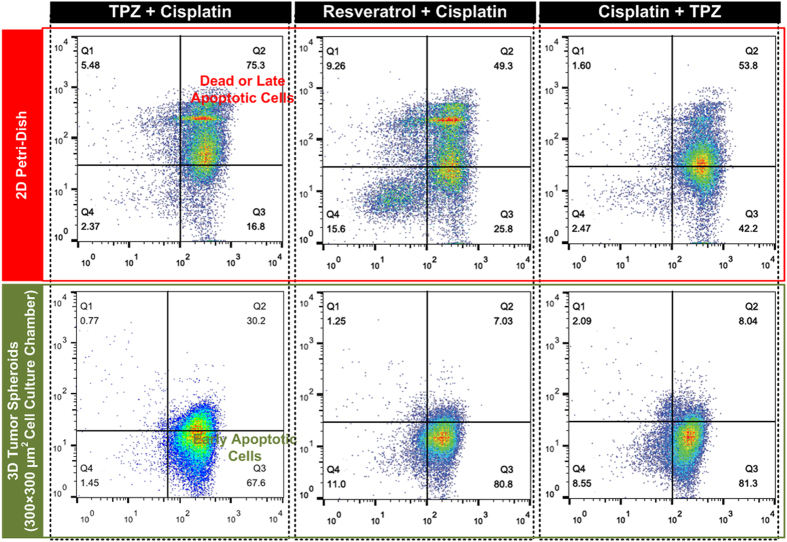
Density plots of flow cytometry analysis on APC-Annexin V and 7-AAD stained HepG2 cells (apoptosis and necrosis assay) cultured in 2D Petri-dish and dissociated from tumor spheroids formed in 300 × 300 μm^2^ (width × length) cell culture chambers after 48-hour treatments of drug combinations.

In contrast, the density plots for drug testing on 3D tumor spheroids show very distinct patterns comparing to the 2D cell culture. For the smaller HepG2 tumor spheroids, the Cisplatin treatment shows the drug leads more cells to early apoptotic stage comparing to the 2D experiment (from 25.3% to 36.7%), and the treated cells have slightly higher dead cell population (32.5% for 2D vs. 38.4 for 3D smaller spheroid). The results suggest that Cisplatin has higher cytotoxicity on the smaller tumor spheroids, and can kill cancer cells in the spheroids with better efficiency. However, for the Resveratrol and TPZ treatment experiments, more cells can stay in live conditions with intact cell membrane integrity (24.4% for Resveratrol and 42.9% for TPZ), which are more than 50% higher than those in 2D experiments. The results also suggest more cells stay in early apoptotic stage and fewer cells are dead in 3D spheroids comparing to the 2D experiments. The results imply Resveratrol and TPZ do not work well and have lower efficiency on the 3D small tumor spheroids. For the larger HepG2 tumor spheroids, the density plots of flow cytometry analysis results show very different patterns comparing to those obtained from the smaller spheroids. All the drug treatment results show more than 80% and 70% reduction in the population of the dead cells comparing to the 2D experiments and the 3D smaller spheroid experiments, respectively. For the Cisplatin treated tumor spheroids, more than half (55.7%) of the cells are kept in early apoptotic stage. In contrast, approximately half (49.1%) of the Resveratrol treated cells and more than half (85.3%) of the TPZ treated cells remain healthy after the 48-hour experiments, which suggest the much greater drug resistance of the larger tumor spheroids. It is noted that a population of cells (23.5% for the smaller tumor spheroids and 25.1% for the larger spheroids) goes to necrotic stage with damaged cell membranes in Resveratrol treatment on spheroid experiments, which is distinct from other drug testing results. Furthermore, the combination drug treatments are also performed in the experiments. Three combinations: Cisplatin + Resveratrol, Resveratrol + TPZ, and TPZ + Cisplatin are tested on the HepG2 cells cultured in 2D monolayer and 3D spheroid (300 × 300 μm^2^ culture chamber) formats for 48 hours. The density plots of the flow cytometry analysis results are shown in Fig. 7. In drug testing on 2D cell culture, all three drug combinations cause more cell death comparing to the single drug treatments. However, for the 3D spheroid drug testing, only about or less than 30% of the cancers cells are dead even the spheroids are treated with relative high concentrations of drugs. Furthermore, more than 60% of cells are kept in the early apoptotic stages for all three drug combinations. Interestingly, the spheroids treated by the drug combinations with Resveratrol do not show the necrotic cell populations as those shown in single drug treatment results in Fig. 6.

## Discussion

Three-dimensional (3D) tumor spheroid possesses great potential as an *in vitro* model to improve predictive capacity for pre-clinical drug testing. The microfluidic device developed in this paper provides a great platform to form and culture a large number (5000) of tumor spheroids for the following analysis. The experimental results confirm that statistically uniform spheroids within a single device as well as different devices can be formed in a simple manner. Furthermore, the cultured spheroids can be harvested from the device with high efficiency by simple manual pipetting. Due to the ample sample population, the harvested spheroids can be further dissociated and labelled for high throughput flow cytometry analysis to achieve statistical investigation of cellular behaviors in 3D cell culture. The high viability after cell culture in the device indicates its great cell compatibility, and confirms the established spheroid culture protocols using the microfluidic devices do not generate additional undesired cytotoxicity.

Because of the great advantages provided by the integrated approach, the device is idea for drug testing and analysis on 3D spheroid culture for pharmaceutical applications. In the experiments, we perform anti-cancer drug testing (Cisplatin, Resveratrol, TPZ, and their combinations) on hepatocyte (HepG2) tumor spheroids for demonstration. The flow cytometry analysis results show very different apoptosis and necrosis patterns between 2D cell culture and 3D spheroids with different sizes. In order to better study the possible factors affect drug efficiency, we also performed the drug tests on 2D cell culture under various oxygen tensions, and the results are shown in Fig. S1 in the supplementary information. From the 2D cell culture results, Cisplatin with concentration in the order of μM has decreased cytotoxicity under lower oxygen tensions. In 3D cell culture, oxygen tension within a tumor spheroid is lower due to limitation of diffusion and consumption by the cells^30^. Therefore, the lower oxygen tensions may make Cisplatin less efficiency in the larger spheroids comparing to smaller spheroids and 2D cell culture. In contrast, cytotoxicity of Resveratrol is not affected by oxygen tensions on 2D cell culture. Interestingly, Resveratrol treatments show very different necrosis and apoptosis assay results between 2D and 3D spheroid cell cultures. In previous studies, Resveratrol has been found to interfere with initiation, promotion, and progression of carcinogenesis^31^. Also, it has been suggested that Resveratrol exert its anti-proliferative effect on HepG2 cells by inducing cell cycle arrest and nitric oxide synthase (NOS) activation^32^. As a result, Resveratrol is a potent inducer of NOx production in the cell culture. The low oxygen tension within the spheroids can greatly prolong the biological lifetime of NO^33^, and may further enhance NO induced necrosis as shown in the flow cytometry analysis^34^,^35^. In the drug testing on 2D cell culture, TPZ shows enhanced efficiency under low oxygen tensions in the concentration range of tens μM, which confirms its hypoxia-activated cytotoxicity on HepG2 cells. It has also been reported that TPZ is more effective on 3D spheroids, even those made of human epithelial carcinoma cells (A431.H9) with relative small diameters, than 2D Petri-dish culture for certain cancer cells, which is likely because TPZ is activated more in spheroids where active oxygen consumption by cells and limits in diffusive oxygen transport creates a hypoxic core similar to actual solid tumors^4^,^36^. However, in our drug testing experiments, the results show the opposite trends. TPZ is less effective in 3D HepG2 spheroids, and the efficiency even reduces when the spheroid diameter increases. The results suggest that the cytotoxicity of TPZ may not solely depend on oxygen tensions in 3D tumor spheroids, which requires more careful studies in the future.

The drug testing results are very similar to the current therapy bottlenecks: chemotherapy has been ineffective in shrinking primary liver cancer, which confirms the great predictive capacity of the developed approach^37^,^38^. Further study of the interactions between the drugs in 3D tumor spheroid formation is also essential in order to improve the treatment efficiency. Consequently, the experimental results of flow cytometry analysis on drug treated cells demonstrate that the cell culture format (2D monolayer vs. 3D spheroid) and spheroid size plays critical roles in drug testing, and also confirm the great capability of flow cytometry in cell analysis. The drug testing experiments demonstrate the practical usage of the developed device to form tumor spheroids with uniform and well-controlled sizes, and the spheroids can be harvested for following flow cytometry analysis due to the ample cancer cell populations without additional instrumentation. With the demonstrated capabilities, more informative flow cytometry assays can be performed on the drug treated tumor spheroids cultured in the microfluidic devices to advance the future anti-cancer therapy development. In addition, the integrated approach developed in this paper provides statistical analysis that is critical for reliable drug testing, which cannot be easily achieved by existing techniques. With the advancement, 3D spheroid cell culture can be exploited as a better *in vitro* model than 2D cell culture to provide great predictive capability before conducting animal experiments.

## Conclusion

In this study, an integrated approach for drug testing and analysis on a number of uniform sized tumor spheroids is developed. In the approach, a two-layered microfluidic device for formation, culture, and harvesting of 5000 uniform-sized hepatocellular carcinoma spheroids (HepG2) is developed. The size of the tumor spheroids can be well controlled by designing the cell culture chamber geometries in the device. Anti-cancer drugs with different modes of actions, including: Cisplatin, Resveratrol, and TPZ, and their combinations are tested on the formed tumor spheroids. Due to the ample cell numbers, the cells dissociated from the spheroids can be exploited for flow cytometry analysis. Flow cytometry is capable to analyze single cells in high throughput manners, and provide statistically informative results for researchers. The analysis results of the drug treated tumor spheroids demonstrate the importance of drug testing on 3D tumor spheroid models. Furthermore, the results also suggest that spheroid size is critical to the cellular responses to the drugs. Therefore, it is essential to well control the spheroid size when performing drug testing. In addition, the density plots obtained from flow cytometry analysis provides more information than that current microfluidic spheroid drug testing platforms can achieve. The informative results has great potential to help the pharmaceutical researchers better understand drug functions on cells in more *in vivo*-like 3D formation. Furthermore, with the simple fabrication and easy operation, the developed microfluidic device is promising for practical usage in biological labs to develop new therapies for cancers and other diseases.

## Additional Information

**How to cite this article**: Patra, B. *et al.* Drug testing and flow cytometry analysis on a large number of uniform sized tumor spheroids using a microfluidic device. *Sci. Rep.* **6**, 21061; doi: 10.1038/srep21061 (2016).

## Supplementary Material

Supplementary Information
