# Supplementary material for: Drug testing and flow cytometry analysis on a large number of uniform sized tumor spheroids using a microfluidic device
Source: Sci Rep. 2016 Feb 15;6:21061. doi: 10.1038/srep21061 (PMC4753452; doi:10.1038/srep21061)
Supplement: Supplementary Information [file srep21061-s1.pdf]

Drug testing and flow cytometry analysis on a large number of uniform sized tumor spheroids using a microfluidic device

Bishnubrata Patra[1,2,+], Chien-Chung Peng[1,+], Wei-Hao Liao[1], Chau-Hwang Lee[1,2,3], and Yi-Chung Tung[1,*]

[1]Academia Sininca, Research Center for Applied Sciences, Taipei, 11529, Taiwan
[2]National Yang-Ming University, Institute of Biophotonics, Taipei, 11221, Taiwan
[3]National Taiwan University, Department of Physics, Taipei, 10617, Taiwan
[*]tungy@gate.sinica.edu.tw
[+]these authors contributed equally to this work

Supplementary Information

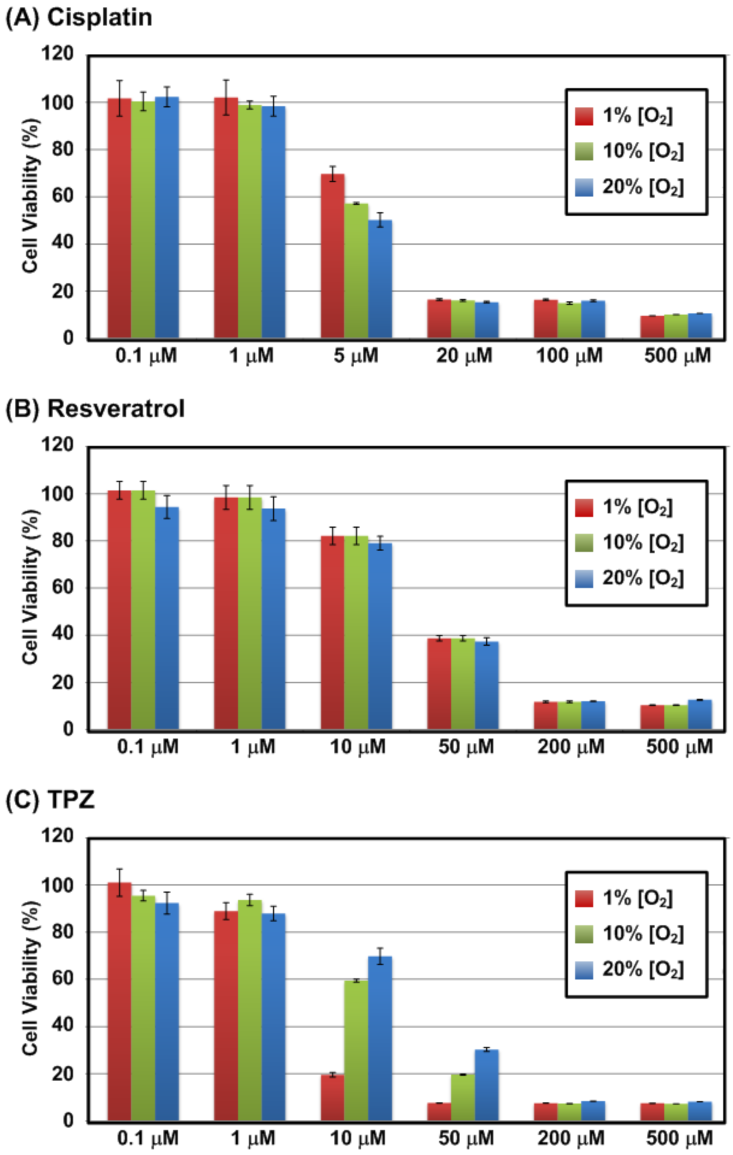

Figure S1. The responses of HepG2 cells cultured in well plates (2D) to (A) Cisplatin, (B) Resvertrol, and (C) TPZ under various oxygen tensions (1%, 10%, and 20%) after 48-hour treatments (n=8). The cell viabilities are characterized by seeding 100 µl of the cell suspension with a density of 50 cells/µl into each well of a 96 well plate. The cells are incubated overnight before the drug treatments. The cells are then treated with three anti-cancer drugs with various concentrations in growth medium under desired oxygen tensions in a cell incubator with oxygen tension control (Heracell 240i, Thermo Scientific Inc., Waltham, MA) for 48 hours. The quantitative cell viabilities are estimated using PrestoBlue Cell Viability Reagent (A-13261, Invitrogen). Following the protocol provided from the manufacturer, 10 µl of PrestoBlue is added to each cell culture well. The plate is then incubated at 37°C and protected from direct light in a cell incubator for 20 minutes. The fluorescence signal from each cell culture well is detected using a commercial microplate reader (Synergy 2, BioTek Instruments, Inc., Winooski, VT) with excitation and emission wavelengths of 530/25 nm and 590/30 nm, respectively.

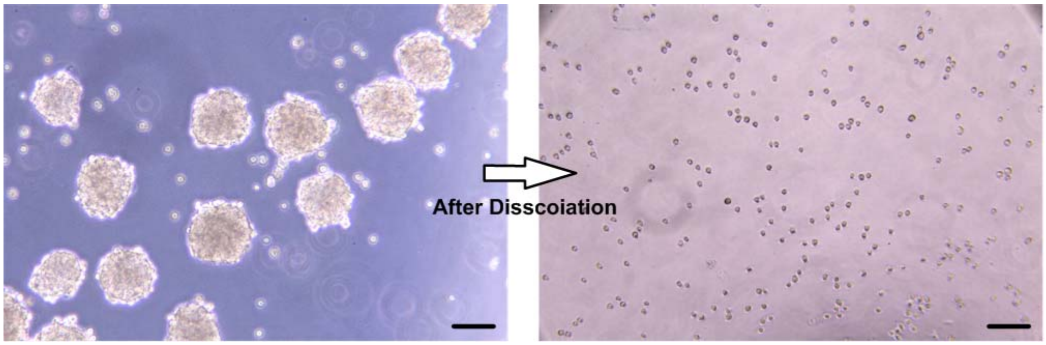

Figure S2. Microscopic photos of the HepG2 spheroids harvested from the microfluidic device, and singles after 5-minute dissociation from the spheroids. In average, more than 93% of the cells can be dissociated into single cell populations. Scale bar is 100 μm.

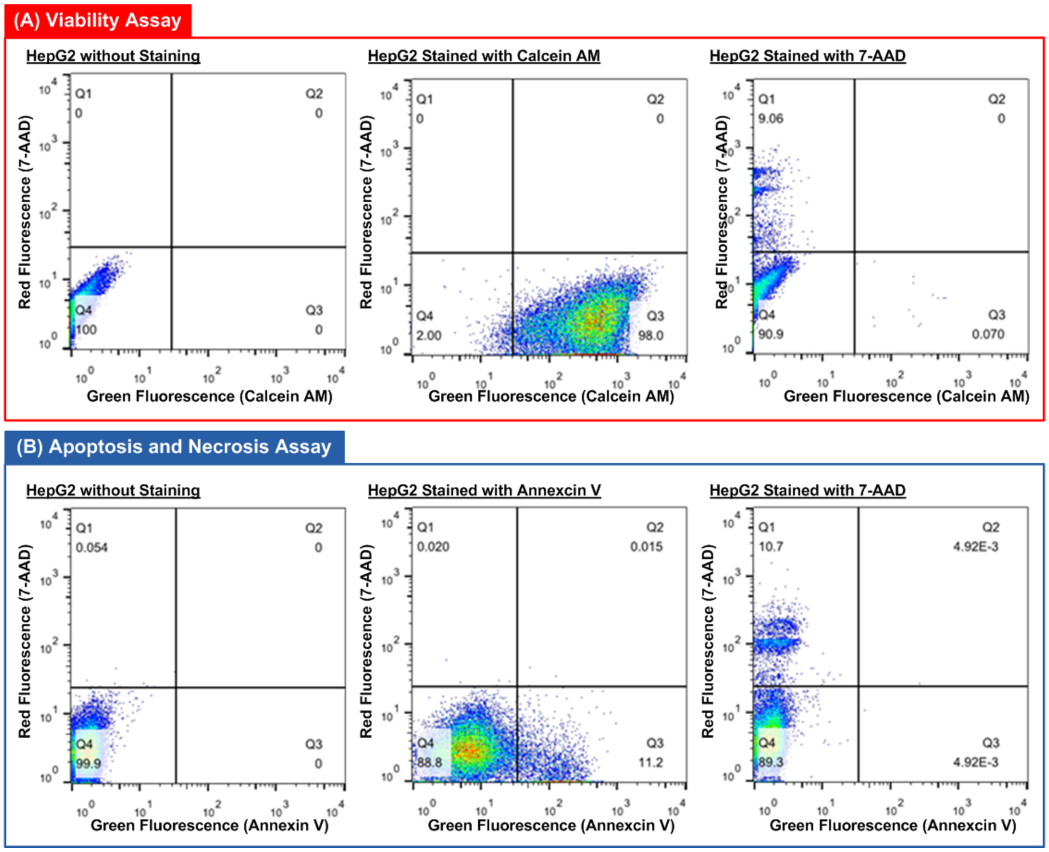

Figure S3. Density plots of gating experiments for (A) Viability assay, and (B) Apoptosis and necrosis assay. Gates in the performed flow cytometry analysis are set by analyzing density plots of the cells (HepG2) without staining and stained by single dyes.

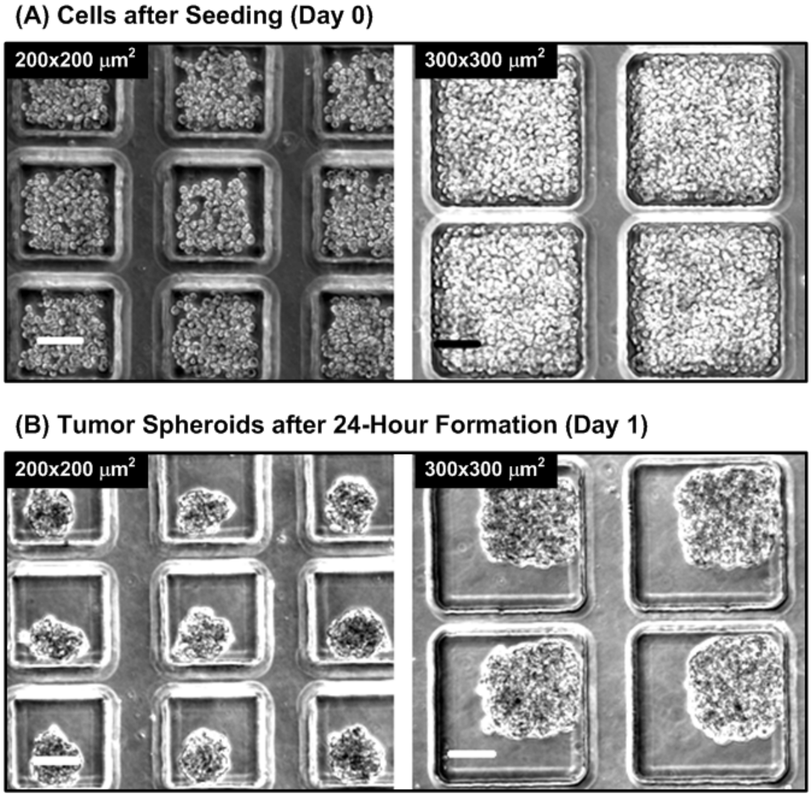

Figure S4. (A) Bright field images of HepG2 cells docked into the cell culture chambers with dimensions (width×length×height) of 200×200×250 μm^3 and 300×300×250 μm^3 due to gravity after cell seeding process. (B) HepG2 cells aggregate to form tumor spheroids within 1-day culture due to their strong cell-cell interaction. Scale bar is 100 μm.
